# Analysis of DNA damage in cells excreted in urine of cervical cancer patients using alkaline comet assay

**DOI:** 10.1186/1755-8166-7-S1-P12

**Published:** 2014-01-21

**Authors:** Avani Patel, Mihir Shah, Pinaki Patel, Trupti Patel

**Affiliations:** 1 School of Biosciences and Technology, Vellore Institute of Technology, Vellore – 632014, Tamil Nadu, India; 2 Sardar Patel University, Vallabh Vidyanagar, Anand, India

## Background

Cancer is one of the most unwanted menaces in the human body. Cancers of lower abdomen are not only life threatening but also painful and survival rate is low. Cervical cancer is second most common worldwide and fifth deadliest in women. It affects about 16 per 100,000 women per year and kills about 9 per 100,000 in a year. In developing countries occurrence rate is 80%. It is possible that there may be no symptom until an advance stage of the cancer is progressed. The single cell gel electrophoresis assay also called comet assay, which is versatile, reliable, powerful, uncomplicated and sensitive technique for detection of DNA damage at the level of individual eukaryotic cell. Understanding the extent of DNA damage in neoplastic cells discarded in the urine of cancer patients through comet assay. Usually normal urine sample have very rare cells. In case of cancer patient the number of cells increases drastically. The type of cells passed in the urine of cancer patient contains mainly leukocytes, some neoplastic cells and some infected tissue cells.

## Materials & methods

The analysis was carried out on 10 subjects having cervical cancer and different levels of DNA damages were seen in cells which were separated from urine. The choice of sample is so, as it is non-invasive.

## Results

The cells have damaged DNA as a result of cancer, which do not have proper binding with histone proteins giving a tail in comet assay which can be easily seen by ethidium bromide staining.

## Conclusions

Comet assay can be used to diagnose the cancer in the suspected patient as an alternative approach to detect the stages of the cancer instead of biopsy and cytology which are painful methods. Early stages of cancer will be detected by non-invasive technique.

